# Feeding Behaviour in the KPC Model of Pancreatic Cancer‐Associated Cachexia: Alteration of Spontaneous and Evoked Feeding Behaviours

**DOI:** 10.1002/jcsm.70262

**Published:** 2026-04-01

**Authors:** Fateema Muzaffar, William F. Colmers, Vickie E. Baracos

**Affiliations:** ^1^ Department of Oncology University of Alberta, Cross Cancer Institute Edmonton Alberta Canada; ^2^ Department of Pharmacology University of Alberta Edmonton Alberta Canada

**Keywords:** appetite, cachexia, calorimetry, indirect, disease models, animal, energy metabolism, feeding behaviour, ghrelin, mice, inbred C57BL, pancreatic neoplasms

## Abstract

**Background:**

The KPC mouse model of pancreatic cancer is gaining use in research, but detailed understanding of ingestive behaviour changes in this and other cachexia models is limited.

**Methods:**

Male C57BL/6 J mice (7 weeks) were maintained under standard feed (PicoLab5L0D) and housing conditions (12:12 h; Light:Dark). Spontaneous feeding was observed in a Comprehensive Lab Animal Monitoring System: meal size, frequency, intermeal intervals and satiety ratio (i.e., time between meals divided by kcal energy in the preceding meal). Feeding was observed at baseline and over time after intraperitoneal injection syngeneic KPC pancreatic adenocarcinoma cells. Separate cohorts of mice were used to study homeostatic feeding responses at baseline and on Day 14 after KPC injection (KPC_14). These responses were fasting‐evoked refeeding, ghrelin injection (1 mg/kg) and lights‐out cue.

**Results:**

Spontaneous food intake declined by 50% by 14 days after tumour injection. At baseline, food intake was observed during 23 of 24 h daily; at KPC_14, hours without any food intake increased from 1 to 12 (*p* ≤ 0.0001). At KPC_14, intermeal intervals increased (*light cycle*: 60.5 ± 11.2 vs. 146.4 ± 19.6 min; *p* < 0.0001; d*ark cycle*: 26.0 ± 3.8 vs. 42.0 ± 5.5 min; *p* = 0.0045), and the satiety ratio tripled (*light cycle*: 23.1 ± 5.9 vs. 69.4 ± 10.5 min/0.1 kcal; *p* < 0.0001; *dark cycle*: 11.3 ± 3.4 vs. 27.1 ± 5.0 min/0.1 kcal; *p* = 0.0028). By contrast, meal size and ingestion rate were unchanged. At KPC_14 mice showed reduced responses to homeostatic feeding cues. After 12‐h starvation, refeeding intake during 4 h was 0.81 ± 0.3 g vs. 0.39 ± 0.19 g at KPC_14 (−52%, *p* = 0.02). Ghrelin response was also blunted 0.66 ± 0.19 g vs. 0.42 ± 0.27 g at KPC_14 (−36%, *p* = 0.005). Mice responded to lights‐out with ~ 4 h of vigorous feeding, ingesting a total of 0.94 ± 0.2 g at baseline; this amount was reduced to 0.55 ± 0.3 g (−41%, *p* < 0.01) at KPC_14, predominantly due to a steep decline in feeding after the end of the first dark hour.

**Conclusions:**

Mice with KPC show response to homeostatic feeding cues; however, these were of diminished amplitude and sustainability. Large increases in intermeal interval and satiety ratio were notable, with mice eating nothing at all for extended periods of time, consistent with enhanced satiety and/or a failure to generate signals sufficient to generate a new feeding event.

## 1 Introduction

Feeding is organized into discrete bouts (meals) that collectively determine total food intake. The dimensions of meals—their size, feeding rate, duration and interval—are regulated by neuronal circuits informed by feedback related to nutrient inflow that shape the progression of ingestion. For example, in the hypothalamus, ghrelin receptor (GHSR)‐expressing agouti‐related protein (AGRP) neurons in the arcuate nucleus (ARC) respond to ghrelin to initiate feeding, while type 4 melanocortin receptor (MC4R)‐expressing neurons in the paraventricular nucleus (PVN) respond to α‐MSH to inhibit feeding. Calcitonin gene‐related peptide (CGRP)‐expressing neurons in the parabrachial nucleus (PBN) of the brainstem regulate meal size. Energy homeostasis is achieved by modulation of meal parameters: Shorter meals are offset by faster feeding rates, larger meals are offset by longer intermeal intervals (IMIs). A starved animal will quickly consume large, frequent meals to restore its energy deficit. This homeostatic modulation is orchestrated by multiple neuronal populations responding to complex feedback, which is humoral and neural (sensory afferents from gut) in nature.

A persistent negative energy balance is a hallmark of cancer cachexia, a debilitating syndrome that severely impacts patient survival and quality of life [1]. Moderate–severely reduced daily food intake has been noted in patients with cancer [2] and in many models of cancer cachexia (Table 1). However, in rodent models, food intake data are only available as aggregate 24‐h measures (or over multiple days), and no meal‐level data have been published. To identify at which steps in the daily feeding pattern nonhomeostatic imbalance arises, feeding must be examined at the level of individual meal parameters. Understanding the complex dynamic of feeding behaviour in cancer cachexia might provide insights into which factors may be targeted to restore energy balance.

**TABLE 1:** Summary of indirect calorimetry use in experimental cancer cachexia models, highlighting food intake reporting and metabolic parameters.

First author (PMID)	Tumour model	Host (strain, sex, age)	Inoculation	System	Food intake	Activity	RER	VO_2_	TEE	Study duration
A. Syngeneic models										
Current study	Pancreatic cancer: KPC Kras^G12D^; P53^R172H;^ Pdx‐Cre^+/+^	C57BL/6J, M, 11–12 week	2–3 × 10^6^	CLAMS	↓	↓	↓	↓	↓	14 days
Liu 2020 (32934774)	Lewis lung carcinoma	C57BL/6, M, 5–7 months	1 × 10^6^ cells, SC	CLAMS	↓^72h^	↓	↓	NR	↔	7–14 days
Kir 2014 (25043053)	Lewis lung carcinoma	C57BL/6, M, 6–10 weeks	5 × 10^6^ cells, SC	CLAMS	↓^12h^	↓	↓	↑	↑	16 days
Choi 2025 (40222332)	Lewis lung carcinoma	C57BL/6, M, adult	5 × 10^6^ cells, IP	Promethion	↓^24h^	↓	↓	↓	↓	14 days
Villars 2017 (28475119)	Colon‐26	CD2F1, M, adult	1 × 10^6^ cells, SC	TSE	↓^24h^	↓	↓	NR	↔	20 days
Kliewer 2014 (25457061)	Colon‐26	CD2F1, M, 5 weeks	1 × 10^6^ cells, SC	CLAMS	↓^24h^	↓	↓	NR	↑	12 days
Tsoli 2012 (22719069)	Colon‐26	BALBc, M, 8–10 weeks	1 × 10^6^ cells, SC	CLAMS	↓^24h^	↓	↓	↔	↓	14 days
Pototschnig 2022 (36351437)	MCA‐207 fibrosarcoma (noncachectic)	C57BL/6 M and F, 10–11 weeks	1 × 10^6^ cells, IM	TSE	↔^24h^	↔	↓	NR		15 days
Pototschnig 2022 (36351437)	CHX‐207 fibrosarcoma (cachectic)	C57BL/6J, M and F, 10–11 weeks	1 × 10^6^ cells, IM	TSE	↓^24h^	↓	↓	NR	↑	15 days
Queiroz 2022 (35941104)	GEMM: KL (Kras^G12D/+^ Lkb1^f/f^)	FVB, M and F, 12–20 weeks	Spontaneous	Promethion	↓^∑ 11d^	↓	NR	NR	↓	12 days
B. Xenograft models										
Bernardo 2020 (32924335)	HT‐1080 fibrosarcoma	ICR‐SCID, F, 8–12 weeks	3–5 × 10^6^ cells, SC & HT	CLAMS‐HC	↓^∑ 14d^	↓	↓	↓	↓	20 days
Lerner 2015 (27239403)	HT‐1080 fibrosarcoma	ICR‐SCID, F, 8 weeks	5 × 10^6^ cells, SC	CLAMS‐HC	↓^24h^	↓	↓	↓	↓	10 days
Suriben 2020 (32661391)	HT‐1080 fibrosarcoma	ICR‐SCID, F, 2–6 months	1 × 10^6^ cells, SC	TSE	↓^24h^	NR	↓	NR	NR	23 days
Wilcox‐Hagerty 2021 (33369797)	MDA‐MB‐231 breast cancer Mets	Athymic nude, F, 5 weeks	1 × 10^5^ cells, HT	TSE	↓^12h^	↓	↓	↓	↓	4 weeks
Clayton 2024 (38659807)	Breast cancer PDOX	NSG, F, 8 weeks	PDX cells, OT	CLAMS	↔^24h^	↔	↔	↓	↓	3 weeks

*Note:* Systems: CLAMS, Columbus Instruments, Columbus, OH, USA; Promethion Metabolic Cage system (Sable Systems, USA); TSE Systems GmbH, Bad Homburg, Germany. Symbols: ↑, increased; ↓, decreased; ↔, no change. Superscripts indicate food intake measurement period: 24 h, 24‐h; 12 h, 12‐h; ∑, cumulative over several days.

Abbreviations: d, day; F, female; GEMM, genetically engineered mouse model; HT, heterotopic; IM, intramuscular; IP, intraperitoneal; M, male; mo., months; NR, not reported; NS, not specified; PDOX, patient‐derived orthotopic xenograft; PDX, patient‐derived xenograft; RER, respiratory exchange ratio; SC, subcutaneous; VO_2_, oxygen consumption.

Pancreatic ductal adenocarcinoma (PDAC) is one of the malignancies most highly associated with cachexia. The KPC model derived from a syngeneic C57BL/6 KRAS^G12D^ P53^R172H^ Pdx‐Cre^+/+^ (KPC) mouse is an established murine model, recapitulating an oncogenic mutation combined with a tumour suppressor mutation common to human pancreatic cancers [2, 3]. KPC induces a wide array of cachexia manifestations, including reduced 24‐h food intake, wasting of adipose tissue, skeletal and cardiac muscle, neuroinflammation, systemic inflammatory responses and haematological and endocrine perturbations. Its reproducible cachexia features and clinical relevance make the KPC model an excellent platform for mechanistic investigations of pancreatic cancer cachexia, resulting in its widespread uptake.

Our objectives were (a) to profile spontaneous behaviour of mice with KPC model using the comprehensive lab animal monitoring system (CLAMS) to permit analysis of fully automated, continuous, high‐resolution feeding data to capture meal‐level food intake and (b) to test responsiveness to evoked feeding behaviours, specifically feeding in response to dark cycle onset (nocturnal feeders), to ghrelin administration and to 12‐h starvation. By resolving which components of feeding behaviour are altered and which remain intact, we may infer the perturbations in underlying regulatory mechanisms driving cachexia‐associated reduction in food intake.

## 2 Methods

### 2.1 Animals, Housing and Feeding

Experimental procedures were approved by the University of Alberta Institutional Animal Care and Use Committee and conducted in accordance with the Canadian Council on Animal Care guidelines. Male C57BL/6J mice (Jackson Laboratory, Bar Harbor, ME, USA), aged 7 weeks, were housed in a low stress facility designed to minimize disruption of rodent feeding behaviour. The unit is controlled for temperature (26°C), light (12 h light 06:00 h:12 h dark), noise, human traffic, vibrations and sudden environmental changes [4, 5]. Access to the unit was restricted to study staff who conducted all study‐related handling and manipulations.

Following 2 weeks of acclimatization, mice were singly housed and habituated for one additional week before assessments. Standard laboratory chow (Laboratory Rodent Diet 5L0D, PicoLab) was provided. While in their home cages, mice had access to custom food hoppers designed to ensure accurate measurement of food intake. Each such hopper had a nested container system, with an outer container collecting spillage and an inner container holding the diet. A spring‐loaded mechanism in the inner container maintained continuous food accessibility by adjusting the diet's position as it was consumed. In the main experiments, mice were transferred to a Comprehensive Laboratory Animal Monitoring System (CLAMS) (Columbus Instruments, Ohio, US). The system employs a Mettler Toledo balance (0.01‐g resolution) to measure cumulative and per‐bout food intake with high precision. The Center Feeder system minimizes spillage and prevents foraging, ensuring accurate quantification of food consumption.

Oxygen consumption (V̇O_2_), carbon dioxide production (V̇CO_2_), respiratory exchange ratio (RER) and heat production (energy expenditure) are continuously assessed via indirect calorimetry in CLAMS. Energy expenditure (kcal/h) was calculated by the CLAMS Oxymax software using the equation: CV * [VO2], where CV (caloric value) is derived from Lusk's equation [6]. Data reported were in accordance with guidelines on reporting indirect calorimetry data [7]. Locomotor activity was continuously recorded using infrared photocells positioned along the X‐ and Z‐axes. Ambulatory activity was quantified when two or more consecutive horizontal beam breaks occurred. All metabolic, locomotor, and feeding data were acquired synchronously, enabling a comprehensive analysis of energy balance and feeding behaviour.

### 2.2 Cancer Cachexia Model

KPC cells (also annotated as KxPxCx) were provided by Dr. Daniel Marks and originally obtained from the laboratory of Dr. Elizabeth Jaffee [8]. Cells were cultured in RPMI 1640 medium (Gibco, #11875‐093) supplemented with 10% foetal bovine serum (FBS, Gibco, 12483‐020) and 1% penicillin–streptomycin (ThermoFisher, SV30010#) at 37°C in a humidified 5% CO_2_ incubator, as previously described by [3]. Cells were harvested, counted and resuspended in phosphate‐buffered saline (PBS). KPC cells were confirmed to be free of mycoplasma and pathogens. Mice were aged 11–12 weeks when injected intraperitoneally at a dose of 2–3 × 10^6^ in 0.4‐mL PBS.

In a pilot study, orthotopic implantation was performed into the pancreatic tail under isoflurane anaesthesia. Subcutaneous buprenorphine was administered preoperatively (0.1 mg/kg) and every 12 h for 36 h postoperatively (0.05 mg/kg) for analgesia. Based on findings of perturbed feeding from the pilot study (See Section 3), intraperitoneal implantation was selected for the main studies.

### 2.3 Experimental Protocol

The main study design included baseline observation of healthy animals, followed by continued observation after injection of KPC cells (within‐animal comparisons). Following a 24‐h acclimatization period in the CLAMS cages, baseline food intake was recorded over three consecutive days. After this baseline assessment, animals were returned to their home cages and 7 days later implanted with KPC tumour cells (or saline) and returned to CLAMS for continuous monitoring until a predetermined endpoint (≥ 50% reduction in daily food intake) was met.

### 2.4 Feeding Pattern Analysis

CLAMS continuously records feeding, capturing each interaction with the food hopper asynchronously. Feeding events were logged with a start time, end time, duration, and weight consumed. A meal was defined as a feeding bout with an intake > 0.03 g and a minimum IMI of 5 min, a threshold we also confirmed through preliminary data analysis to optimize accuracy while minimizing noise [9, 10]. Quantified feeding parameters included meal size (kcal), meal duration (min), IMI (min), satiety ratio (SR) (min/0.1 kcal, calculated as the IMI divided by energy intake at the preceding meal) and ingestion rate (kcal/min, calculated as meal size divided by meal duration) (Figure 3A).

### 2.5 Evoked Feeding Responses

Feeding responses were assessed in separate cohorts of mice, each both at baseline and after 14 days of tumour growth. Spontaneous dark cycle feeding and fasting‐evoked refeeding were assessed in the CLAMS system, and ghrelin‐induced feeding was evaluated in home cages. For Feeding Response at Dark Cycle Onset, food intake during the first 4 h of the dark cycle was evaluated. For starvation‐Evoked Feeding, mice underwent a 12‐h dark cycle starvation (food withheld, water available ad libitum). Food access was returned at light onset (06:00). For Ghrelin‐Evoked Feeding, mice were administered ghrelin (1 mg/kg, *i.p*.; PUBCHEM: 16139313; AnaSpec, CA, USA) or vehicle (sterile saline) at the beginning of the light cycle (08:00 h).

### 2.6 Human Data

In a recent study, 12 253 patients with different primary cancers at risk for cancer‐associated weight loss were evaluated for food intake (normal, moderately or severely reduced), and the association of reduced food intake with weight loss and mortality was assessed [11]. Here, we conducted a subanalysis of the published data set, comparing patients with pancreatic cancer to other primary cancers regarding food intake impairment and the prevalence of symptoms associated with reduced appetite (anorexia, early satiety, pain, nausea and vomiting).

### 2.7 Statistical Analysis

Analyses were performed using Prism 8.0 (GraphPad Software, San Diego, CA). Data are presented as mean values ± SEM, unless otherwise specified. Between‐group comparisons of daily food intake were analysed using a two‐way repeated measures ANOVA. Unpaired comparisons were made between groups, whereas paired comparisons were made within each animal, comparing observations before and after tumour implantation. For meal‐related parameters, linear mixed‐effects models were used with animals as a random intercept to account for repeated measures, individual variability and unequal numbers of meals between observations. For temporal data, hourly comparisons between baseline and KPC Day 14 were conducted using paired *t*‐tests at each time point. Fisher's exact test was used for a test of proportions. Results were considered significant at *p* < 0.05. All studies were replicated in cohorts of 6 or 7 animals per treatment group due to the capacity of the CLAMS system (12 cages). Replicates were pooled, and the total number of animals per group is indicated in each table and figure.

## 3 Results

### 3.1 Preliminary Findings: Selection of Route of Implantation

Intraperitoneal (IP) and orthotopic (OT) routes of implantation produce similar histopathological features and manifestations of cancer cachexia. However, the procedures differ markedly in invasiveness: IP implantation involves a simple injection of tumour cells into the peritoneal cavity without anaesthesia, whereas OT implantation requires surgical exposure of the pancreas and direct injection into pancreatic tissue under anaesthesia, along with preoperative and postoperative analgesia.

We conducted a pilot study to compare the effects of sham IP and OT procedures on food intake. Sham OT procedures (saline was injected) caused a transient reduction in food intake, with a 40% decrease on day 1 (*p* = 0.002), a 19% decrease on day 2 (*p* = 0.04) and a return to baseline by day 3 (Figure S1). These effects likely reflect the combined influence of surgical stress, anaesthesia and analgesics. Sham IP injections had no effect on food intake. Based on these findings, we selected the less invasive IP injection for all subsequent studies. IP implantation predominantly localized tumours to the pancreas with some extra‐pancreatic disease development, preserving cachexia manifestation while minimizing surgical stress and postoperative recovery.

#### 3.1.1 Meal‐Level Behaviour of the Animals in the CLAMS Was Stable

**FIGURE 1:**
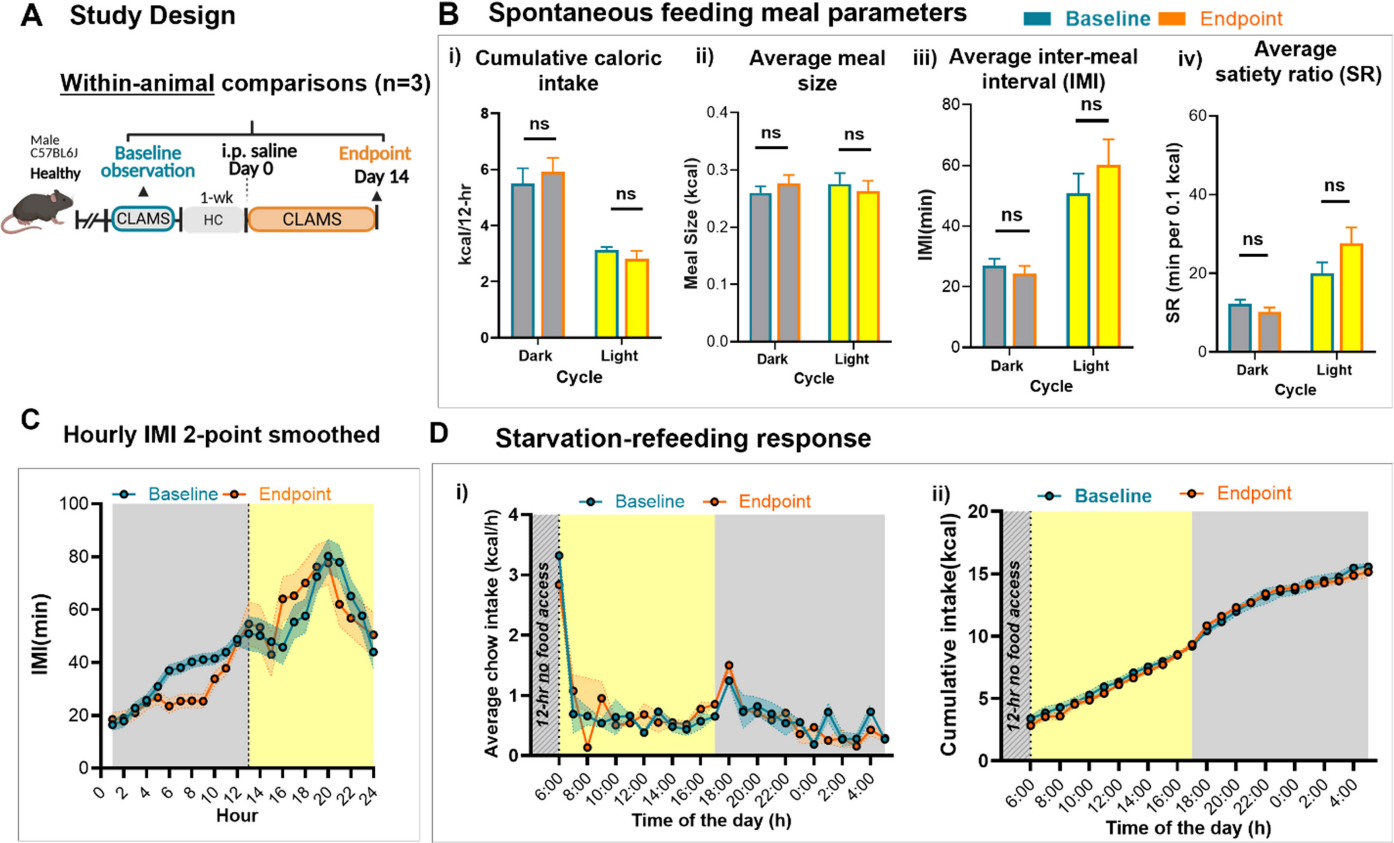
Spontaneous meal‐level feeding and starvation–refeeding response of healthy animals recorded in the CLAMS system was stable over the study duration. (A) Study design; within‐animal comparison of meal parameters in saline‐injected control mice (Sham; *n* = 3) at Baseline vs. study endpoint. (B) Spontaneous meal parameters: (i) cumulative caloric intake, (ii) meal size, (iii), intermeal interval (IMI) and (iv) satiety ratio (SR). Linear mixed‐effects model (LMM) with ‘observation’ (baseline vs. Day 14) as a fixed effect and ‘animal’ as a random intercept found no significant effects in either light or dark cycle (*p* > 0.05 for all). (C) IMI trend over 24 h as a 2‐point smoothed, variability (± SEM) is shown as shaded bands. Baseline: Light phase ± 6.5 min, Dark phase ± 2.32 min. Endpoint: Light phase ± 8.33 min, Dark phase ± 2.6 min. (D) Starvation‐refeeding response: (i) hourly and (ii) cumulative refeeding response to 12‐h dark cycle food restriction; food access was returned at lights on (6:00), and animals have access to water during the food restriction.

To justify a repeated‐measures design comparing meal‐level feeding within animals across health and disease states, we first documented the stability of feeding behaviour in healthy mice housed in the CLAMS system over time (Figure 1A). This analysis confirmed no significant alteration in total intake, meal size, IMI or SR (Figure 1B,C). Furthermore, the response to a 12‐h starvation‐refeeding was superimposable between baseline and the end of study. These findings indicate that extended CLAMS housing does not alter the meal‐level behaviours under investigation in our model.

### 3.2 Evaluation of Body Weight and Tumour Mass

Sham mice started at 26.9 ± 1.4 g and increased to 27.6 ± 1.3 g, corresponding to an absolute gain of +0.65 ± 0.58 g (+2.4% relative to baseline). KPC mice started at 26.8 ± 0.8 g and showed an absolute weight change of −1.98 ± 0.2 g over 14 days (−7.3% relative to baseline) (Figure S2A). Body weight values at Day 14 include tumour mass in the tumour‐bearing group.

By Day 14 postinjection, IP implantation of tumour cells resulted in a consistent disease development. The major tumour mass localized to the tail of the pancreas, and there were additionally small (< 2 mm) sporadic extra‐pancreatic nodules and ascites. The combined pancreatic/tumour mass was 1.64 ± 0.44 g, compared with 0.29 ± 0.05 g in sham controls (*n* = 6 per group) (Figure S2B). Extra‐pancreatic nodules were observed in all animals to a similar degree but not quantified. Ascites were present in all tumour‐bearing mice at volumes below the threshold for reliable quantification (< 0.2 mL).

### 3.3 Overall Changes in Food Intake Across Tumour Progression

**FIGURE 2:**
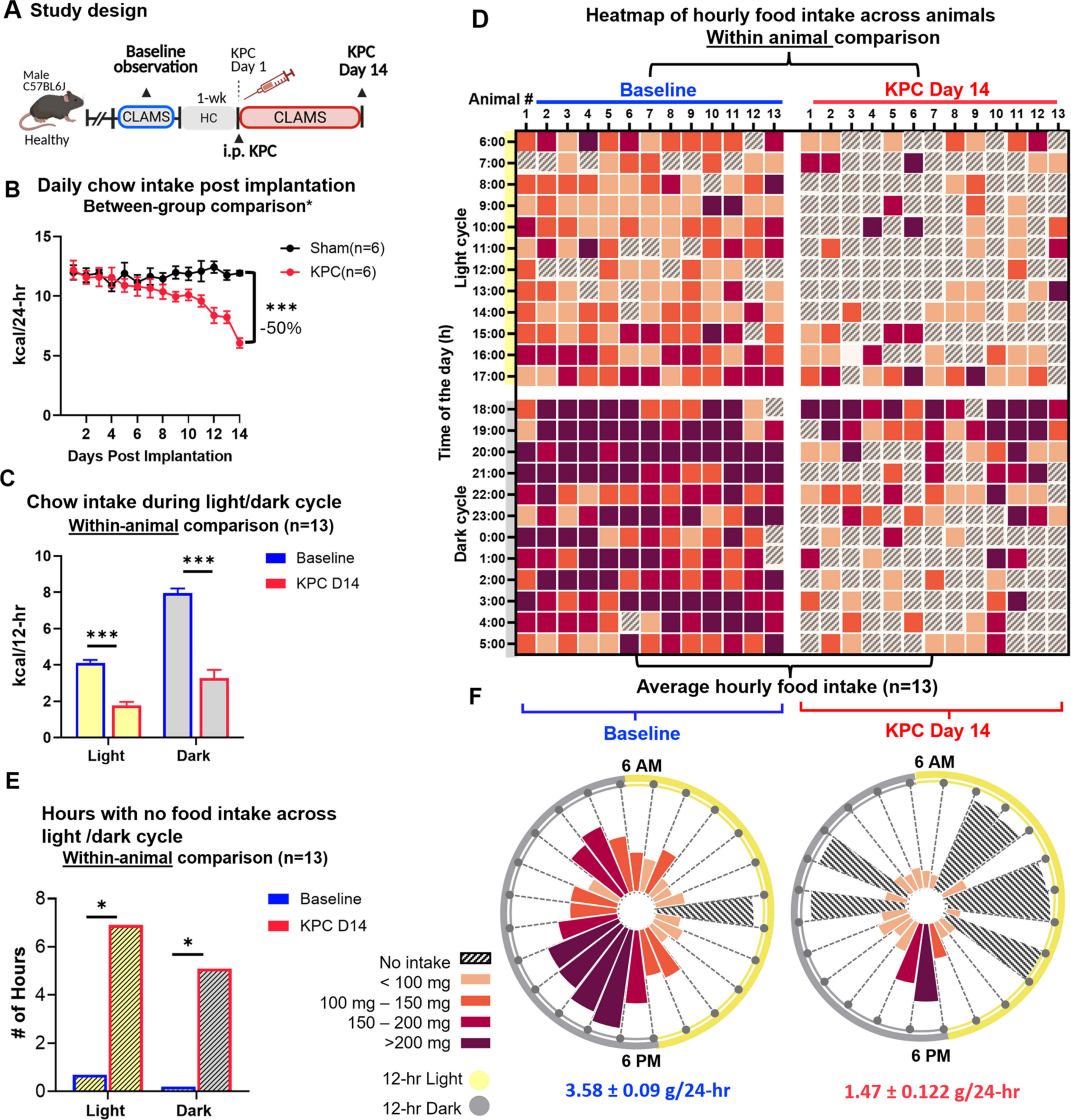
Progressive decline and disruption of feeding patterns in KPC tumour‐bearing mice at day 14 postimplantation relative to their pre‐implantation baseline. Data from *n* = 06 per group presented in Panel B; data from *n* = 13 presented in Panels C–E: (A) Study design: Healthy mice were acclimated to individual housing and underwent a 3‐day baseline feeding assessment in the CLAMS (Comprehensive Lab Animal Monitoring System) system. Animals were returned to home cages and after ∼7 days were injected intraperitoneally (i.p.) with KPC cells, then returned to CLAMS until Day 14 postimplantation (KPC D14), defined as the point of 50% reduction in 24‐h food intake. (B) Daily food intake between‐group comparison of food intake decline in KPC tumour‐bearing mice and saline‐injected sham mice (*n* = 6 per group). (C) Within‐animal comparison of average 12‐h food intake during the light (6 AM to 6 PM) and dark (6 PM to 6 AM) cycles, comparing Baseline to Day 14. (D) Heat map visualizing within‐animal change in hourly food intake from baseline to KPC Day 14. Each cell represents the total food intake for an induvial mouse with each 1‐h time bin. (E) Number of hours with no food intake during light and dark cycles at baseline and Day 14 (F) Radial plots of within‐animal change in 24‐h feeding patterns from Baseline to Day 14. Total 24‐h consumption (g/24‐h) is noted. Statistical analyses were conducted repeated measures ANOVA (B), paired *t*‐tests (C), or one‐tailed Fisher's exact tests (E). *p ≤ 0.001.

KPC‐implanted mice exhibited an exponential fall in food intake from Days 6 to 14, while sham‐injected mice maintained stable food intake throughout 14 days (Figure 2A). The 24‐h food intake on Day 14 in KPC mice (KPC_14) was 6.07 ± 1.0 kcal, a 50% reduction compared to sham mice ≤ 0.0001, *n* = 6 per group (Figure 2B). Hereafter, results are presented as within‐mouse comparisons between each animal's baseline (healthy) behaviour and 14 days after KPC implantation (KPC_14). At baseline, mice consumed 4.12 ± 0.2 kcal/day during the light cycle and 7.9 ± 0.3 kcal/day during the dark, accounting for 34.2% and 65.8% of total daily intake (Figure 2C), respectively. KPC resulted in a uniform reduction of ~ 50% of light and dark cycle feeding. By KPC_14, light cycle intake was 2.37 ± 0.2 kcal/day, while dark cycle intake declined to 4.74 ± 0.4 kcal/day (*p* ≤ 0.0001).

To visualize the diurnal rhythm of feeding, we represented 24 × 1 h binned feeding data as a heat map at baseline and at Day 14 within animals (Figure 2D). Healthy mice consumed food during 23 of the 24‐h day, while at KPC_14, the number of hours with no feeding increased to 12 (7 h in light and 5 h in dark), compared to only 1 h during light at baseline (Figure 1E; Fisher's exact test, *p* < 0.0004). The heatmap permits observation that mice completely stopped eating for extended periods with some intra‐individual variability (ranging from 2 to 8 consecutive hours). Radial plots (Figure 2F) show an average 24‐h feeding patterns across all animals at baseline (left) and KPC_14 (right).

### 3.4 Meal‐Level Parameters

**FIGURE 3:**
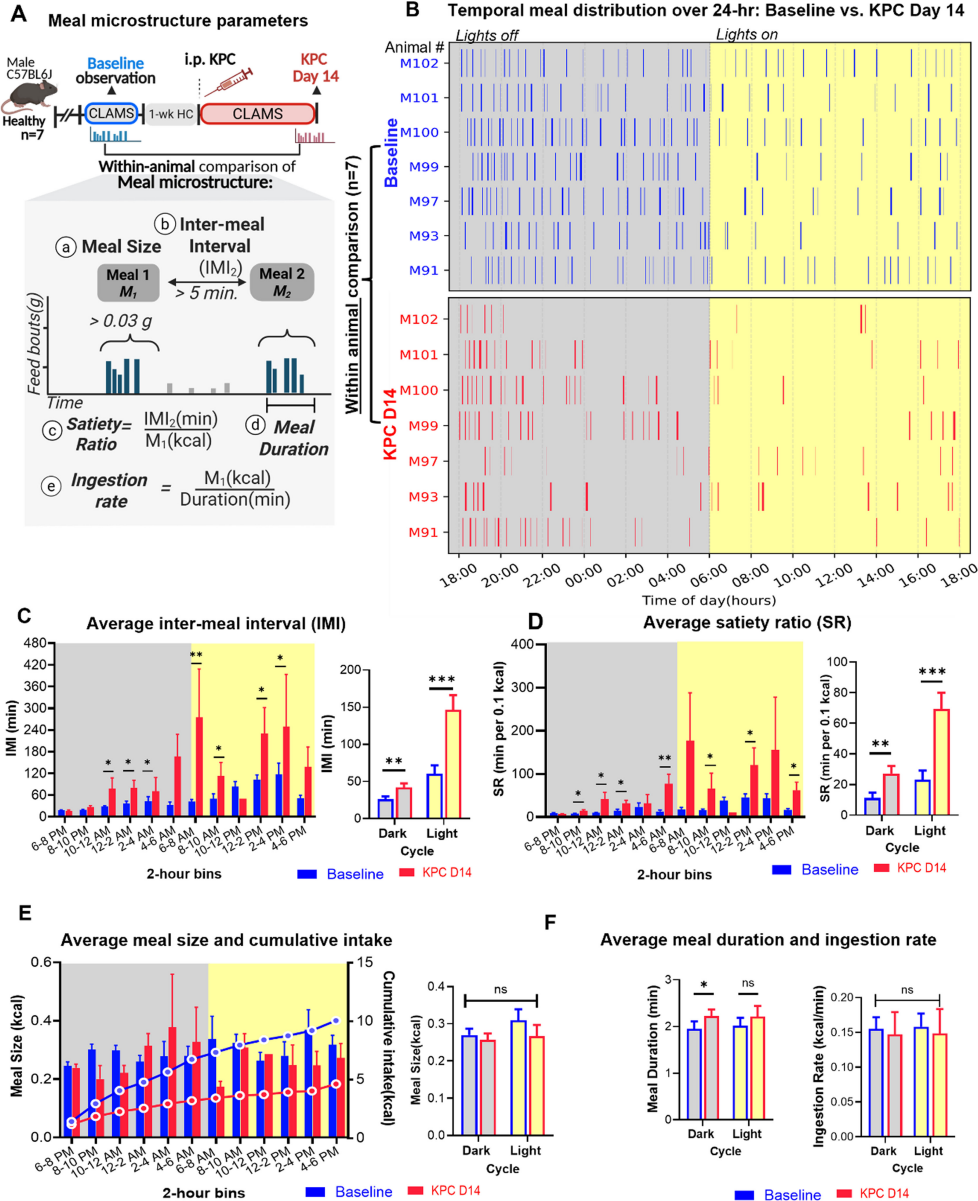
Meal‐level parameters changes in KPC mice at Day 14 postimplantation relative to healthy baseline. Data from (*n* = 7) animals are represented (Panels A–F). (A) Study design and the schematic of meal parameters: intermeal interval (IMI), satiety ratio (SR), meal size, meal duration and ingestion rate. (B) Raster plots of meal distribution over 24 h at baseline and Day 14. (c) IMI. (d) SR. (e) Meal size. (f) Meal duration and ingestion rate. Bar plots showing estimated marginal means (± SEM) derived from linear mixed models for each variable during light and dark cycles at baseline and endpoint (*p < 0.05, **p < 0.01, ***p < 0.0001).

To structure the CLAMS food intake data, we utilized the behavioural definition of “meals” as periods of feeding and drinking, separated by noneating intervals (> 5 min) [10]. Since food intake was continuously monitored, using meals as a unit allowed us to segment the data and focus on the dynamics of feeding patterns. We compared meal microstructure within animals at baseline and at KPC_14 (Figure 3A).

KPC‐implanted mice exhibit marked reductions in meal frequency, prolonged IMIs and SR. Raster plots of feeding events (Figure 3B) revealed distinct differences in the temporal distribution of meals between mice at baseline and on KPC_14. At baseline, feeding occurred throughout the 24‐h cycle, with a higher meal frequency during the dark. The overall average number of meals was 22 in the dark and 10 meals in the light. Meal frequency was reduced at KPC_14 (11 meals in the dark vs. 5 meals in the light cycle, *p* < 0.0001). The IMI was increased at KPC_14 compared to baseline (Figure 3C). During the dark, IMI increased from a baseline mean of 26.0 ± 3.78 min to 42.02 ± 5.5 min (*p* = 0.0045; *n* = 7). During the light, the IMI increased from 60.5 ± 11.2 min at baseline to 146.4 ± 19.6 min (p < 0.001; n = 7).

The SR increased at KPC_14 (Figure 3D). Overall, during the dark cycle, the baseline SR was 11.3 ± 3.4 min/0.1 kcal, compared to 27.1 ± 5.0 min/0.1 kcal at KPC_14 (*p* = 0.002, n = 7). During the light cycle, the baseline SR was 23.1 ± 5.9 min/0.1 kcal, which increased to 69.4 ± 10.5 min/0.1 kcal at KPC_14 (*p* < 0.001, n = 7). Increased SR was especially prominent in the early part of the light cycle, increasing over 10‐fold at KPC_14 (Figure 3D). Between 6:00 and 8:00 AM, the SR rose from 17.9 ± 4.9 min/0.1 kcal at baseline and 17.8 ± 11.1 min/0.1 kcal at KPC_14. During midday (12:00–2:00 PM), the SR was 45.6 ± 7.6 min/0.1 kcal. at baseline and 120.4 ± 3.9 min/0.1 kcal.

#### 3.4.1 Some Aspects of Meals Were Minimally or Not Affected by Pancreatic Cancer

Meal size in mice remained comparable during the dark cycle between baseline and KPC_14 (0.277 ± 0.028 kcal vs. 0.272 ± 0.052 kcal, *p* = 0.42) (Figure 3E). While meal duration during the dark showed a slight increase (1.94 min → 2.22 min; +0.28 min, *p* = 0.049), there were no differences in either meal duration (light cycle) or ingestion rate between baseline and KPC_14 (Figure 3F). This consistency suggests that the reduced food intake in KPC‐implanted mice is driven primarily by decreased meal frequency.

### 3.5 Attenuated Responses to Homeostatic Feeding Cues

**FIGURE 4:**
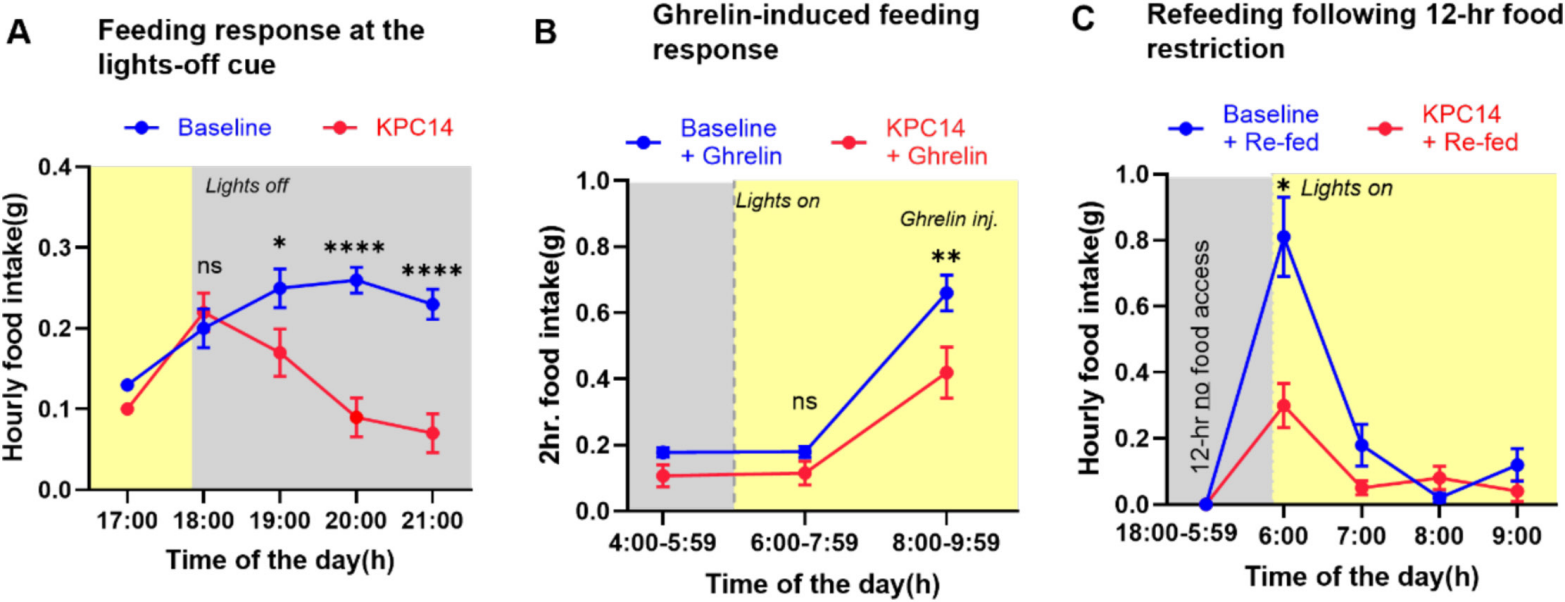
Attenuated acute feeding response to homeostatic feeding signals in KPC tumour‐bearing mice at Day 14 postimplantation relative to their pre‐implantation baseline. (A) Hourly cumulative food intake during dark cycle transition (*n* = 13; grey shading indicates dark cycle). (B) Two‐hour food intake following I.P. ghrelin administration (1 mg/kg; *n* = 12). (C) Refeeding response after 12‐h dark cycle food deprivation, measured at light cycle onset (*n* = 7; yellow shading indicates light cycle). Data from CLAMS presented as mean ± SD Statistical analysis performed using paired *t*‐tests (*p < 0.05, **p < 0.01, ***p < 0.0001, ns: not significant).

At baseline, feeding behaviour exhibited a pattern typical of this nocturnal feeding species, with robust feeding triggered by the lights‐off cue (Figures 2D and 2E). A closer analysis of feeding after lights‐out appears in Figure 4A. During the first dark hour, mice consumed identical amounts of food at baseline and at KPC_14 (0.20 ± 0.09 g vs. 0.22 ± 0.09 g). After that first hour, food intake was sustained when mice were healthy but faltered after over the next 3 h at KPC_14 (*p* = 0.0002, *n* = 13). KPC also attenuated the feeding response to ghrelin (Figure 3B) from 0.66 ± 0.19 g over a 2‐h period to 0.42 ± 0.27 g (*p* = 0.005, *n* = 12). Starvation‐induced refeeding was also blunted at KPC_14 (Figure 4C). Following a 12‐h starvation during the dark cycle, healthy mice consumed 0.81 ± 0.3 g in the first hour food were made available at the beginning of the subsequent light cycle. By contrast, at KPC_14, the same mice consumed less, averaging only 0.39 ± 0.19 g (*p* = 0.02, *n* = 7).

### 3.6 Secondary Objectives: Energy Expenditure and Activity

**FIGURE 5:**
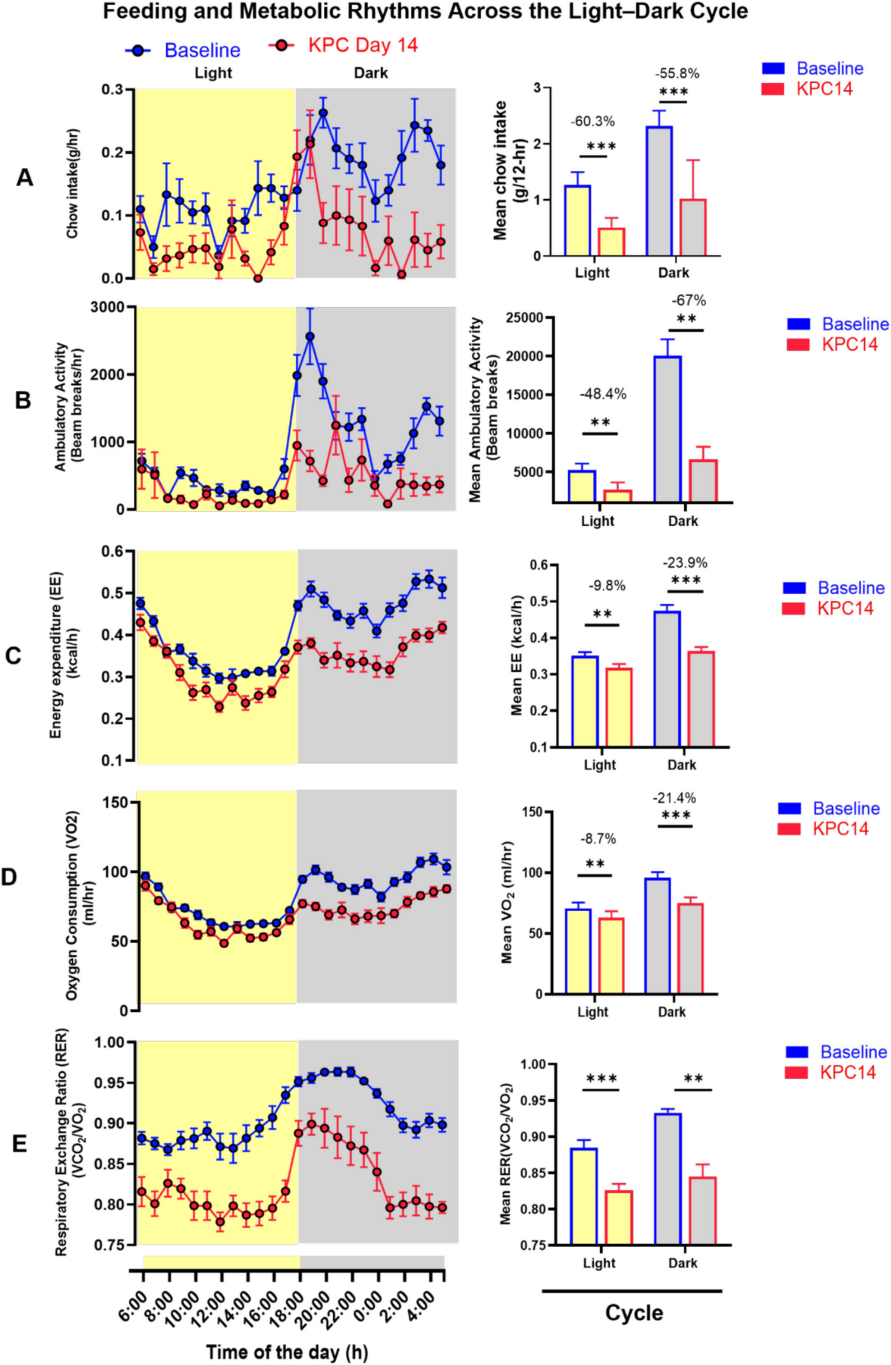
Food intake, energy metabolism and activity at Day 14 postimplantation in KPC tumour‐bearing mice relative to their pre‐implantation baseline. Data from (*n* = 7) animals are represented (Panels A–D). Temporal profiling of feeding and metabolism in mice at baseline and 14 days following KPC tumour implantation. (A) Chow intake, (B) ambulatory activity, (C) heat production, (D) oxygen consumption, and (E) (VO_2_) respiratory exchange ratio during light and dark cycles at baseline (blue) and Day 14 posttumour implantation (red). Left panels show 24‐h temporal profiles beginning at 6:00 AM; right panels display mean values for light (6:00–18:00) and the dark (18:00–6:00) cycle. Grey shading denotes the dark cycle. Data are presented as mean ± SEM Statistical analysis was performed using paired t‐tests for light/dark comparaisons (*p < 0.05, **p < 0.01, ***p < 0.001). *Note*: See tumour and body weight data in Figure S2.

CLAMS systems permit integrated measures of intake, activity and expenditure of energy, and data of this type exist for some cachexia models, but not KPC until our study (Table 1). Based on continuous measures in the CLAMS system, and again using within‐animal paired comparisons, these adaptations were observed both at baseline and at KPC_14 (Figure 5). Under baseline conditions, mice exhibited a diurnal pattern in ambulatory activity and energy expenditure; these parameters increased sharply at lights‐out. Overall dark cycle activity was 3.8‐fold higher than in the light (Figure 5A). Energy expenditure (Figure 5B) and VO_2_ (Figure 5C) followed a similar pattern as activity, with sustained increases during the dark cycle. By KPC_14, ambulatory activity had declined significantly, with a 67% decrease in dark cycle movement and a 48% decline in the light cycle. Energy expenditure and V̇O2 were lowered in the light cycle by 8%–9% and were lowered in the dark by 21%–23%. The respiratory exchange ratio (RER—Figure 5D) exhibited a pattern synchronized with feeding. Higher RER values during the dark cycle reflect predominant carbohydrate oxidation, while lower light cycle RER values indicate enhanced reliance on lipid metabolism.

**FIGURE 6:**
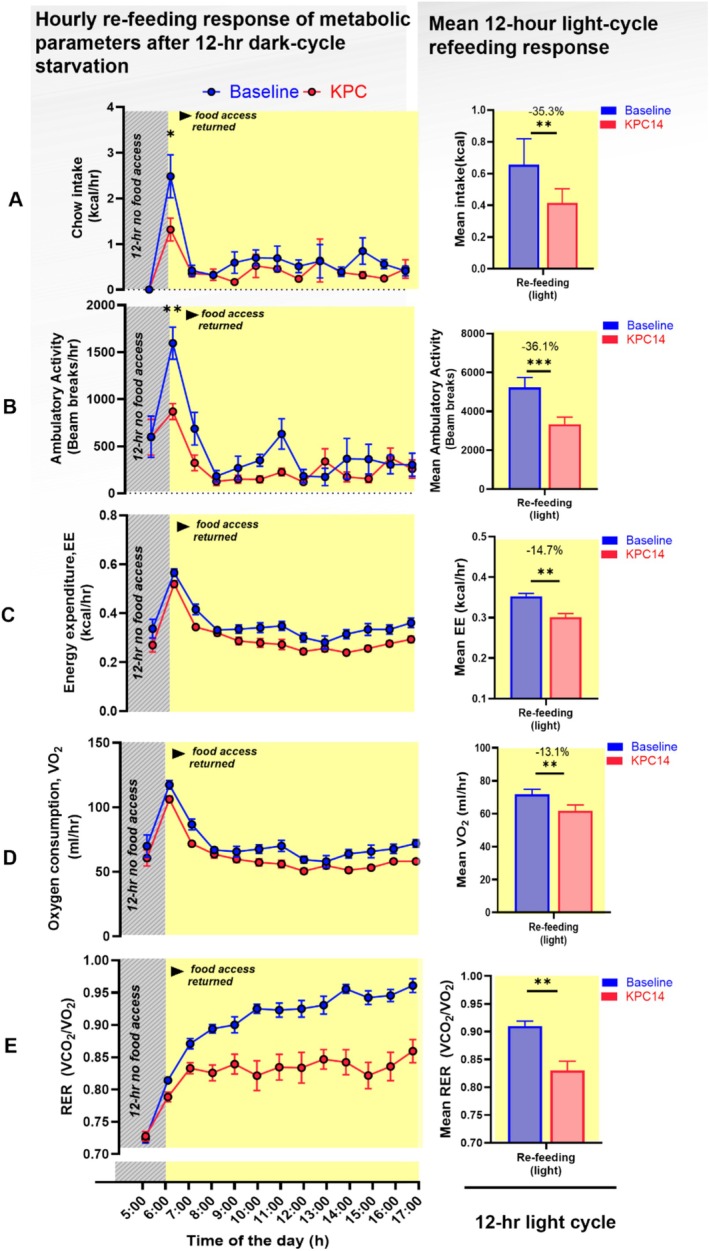
Food intake, energy metabolism and activity responses to 12‐h starvation‐refeeding at Day 14 postimplantation KPC tumour‐bearing mice relative to their pre‐implantation baseline. Data from (*n* = 7) animals are represented (Panels A–‐E). Paired comparisons within animal between baseline and KPC Day 14 following 12‐h dark cycle starvation (18:00–6:00) followed by ad libitum refeeding at the start of the light cycle (6:00). (A) Hourly food intake. (B–E) Corresponding hourly profiles of ambulatory activity (B), heat production (C), oxygen consumption (VO_2_, D) and respiratory exchange ratio (RER, E). Left panels display 24‐h temporal profiles; right panels show the 12‐h light cycle average for each parameter. Yellow shading indicates the light cycle. Data (mean ± SEM) were collected via CLAMS. Within animal comparison at baseline (blue) and KPC Day 14 (red). Paired *t*‐tests were used for within‐cycle comparisons (*p < 0.05, **p < 0.01, ***p < 0.001).

Metabolic responses to refeeding after 12 h of starvation in the dark cycle are illustrated in Figure 6. At baseline, renewed access to food provoked an immediate spike in food intake and activity as well as a brief spike in VO_2_ and energy expenditure. At KPC_14, mice retained the pattern (i.e.*,* spike in intake and activity in the first hour of refeeding) (Figure 6A,B) but with significantly reduced magnitude. During the 12 h following renewed food access, the presence of KPC was associated with reductions in intake, activity, VO_2_, energy expenditure and RER compared with their baseline values.

### 3.7 Symptoms of Anorexia and Satiety in Patients With Pancreatic Cancer

Several aspects of translational relevance have been established in the KPC model [12, 13, 14]. Patients at risk for cachexia are profiled for their level of food intake and symptoms affecting food intake [11]. We used the data set from Martin et al. [11], to compare patients with pancreatic cancers to those with other primary malignancies (Table 2). Patients with pancreatic cancer showed similarities with the mice implanted with KPC, including a high prevalence of severely reduced dietary intake and among the tumour sites the highest prevalence of both lack of appetite and early satiety.

**TABLE 2:** Food intake categories and nutrition impact symptoms across primary tumour types in patients with advanced cancer (pooled multicentre cohort [9]).

	Food intake % of patients^a^			Nutrition impact symptoms % of patients^a^					
Primary	Normal	Moderate decrease	Severe decrease	Anorexia	Early satiety	Nausea	Vomit	Pain	Dysphagia
Tumour (*n*)									
Pancreas (359)	11.7%	59.6%	28.7%	56.2%	46.9%	33.5%	15.2%	41.90%	5.60%
Gastroesophageal (301)	11.6%	38.5%	49.8%	41.8%	37.8%	29.6%	23.0%	35.40%	41.20%
	NS	0.001	0.001	0.001	0.06	NS	0.016	NS	0.001
Colorectal (992)	31.5%	57.0%	11.6%	22.8%	19.0%	11.0%	4.2%	16.40%	2.30%
	0.001	NS	0.001	0.001	0.001	0.001	0.001	0.001	0.001
Head and neck (1074)	52.6%	24.9%	22.4%	25.0%	16.0%	8.0%	3.1%	36.40%	36.50%
	0.001	0.001	0.018	0.001	0.001	0.001	0.001	NS	0.001
Lung (1782)	35.4%	46.3%	18.3%	40.6%	25.2%	16.7%	6.5%	26.20%	13.20%
	0.001	0.001	0.001	0.001	0.001	*0.001*	0.001	0.001	0.001
Breast (258)	37.5%	46.8%	15.7%	48.5%	35.8%	43.8%	19.6%	35.40%	8.70%
	0.001	0.002	0.001	NS	0.06	0.009	NS	NS	NS

Abbreviation: NS, not significant.

^a^ In each column Chi‐square *p*‐value for difference from pancreas.

## 4 Discussion

While declines in daily food intake are noted in many rodent models of cancer cachexia, aggregate 24 h measures fail to capture the dynamic regulation of feeding that underlies meal control. Here, we provide the first comprehensive meal‐level characterization of feeding in any established rodent cachexia model. Large increases in IMI and SR occurred, with mice eating nothing for extended periods of time, consistent with enhanced satiety and/or a failure to generate signals sufficient to generate a new feeding event. In conditions of low or no food intake, mice with KPC turn to lipid oxidation but are poorly equipped to spend prolonged periods in fasting metabolism, because pancreatic cancer severely impairs ketogenesis and the muscle—sparing effect of ketones is lost [15]. Despite reduced overall intake, meal size, meal ingestion rate and initial responses to canonical hunger cues were relatively well preserved in mice with KPC, but appetitive drive was reduced. Dark cycle feeding faded prematurely, a ghrelin response was present but muted, and refeeding after starvation ended before the caloric deficit of the preceding night had been made up. Each physiological or pathological state associated with reduced food intake seems likely to be characterized by unique perturbation of meal‐level feeding. It is already known that specific suppressors of feeding (e.g., GLP‐1, activation of POMC or CGRP neurons, CCK, PYY, leptin) reduce intake through specific alterations in meal size, rate of feeding and/or interval, rather than by uniform suppression across all stages of ingestion (summarized in Table S1). Further studies using our analytical framework to describe feeding behaviour are warranted to determine whether cancer is associated more broadly with the behaviours noted here, or whether meal‐level feeding is cancer‐type specific. We suggest that meal‐level parameters should be prioritized, to identify specific deficits within feeding regulation. In KPC, restoration of IMI, as well as identification of high potency activators of appetitive drive, would be pertinent.

KPC‐induced alterations in feeding behaviour can be considered in context of recent findings on regulation of food intake by specific brain regions and by afferent information to these brain regions that are perturbed in the tumour bearing state. Progressive worsening of negative energy balance suggests mechanisms and mediators which potently suppress feeding. Neurocircuits in the brainstem can override homeostasis and impose negative energy balance. Physiologically, the nucleus tractus solitarius (NTS), area postrema (AP) and parabrachial nucleus (PBN) integrate visceral signals and serve critical protective functions by preventing overfeeding through detection of gastric distention and osmolar overload, halting intake of toxic compounds, inducing emesis and conditioning aversive memories [16]. Within the PBN, output from neurons in the external lateral subdivision expressing CGRP causes anorexia, and their chronic activation is sufficient to induce starvation [17, 18]. This neuronal population has been studied in one rodent cachexia model to date: Campos et al. demonstrated constitutive activation of PBN‐CGRP neurons in mice with Lewis Lung Carcinoma (LLC), and ablation of these neurons with tetanus toxin prevented/reversed LLC‐induced reduction of food intake [19]. Inputs to the AP/NTS include vagal signals of distension and malaise, as well as anorectic peptides including GDF‐15. GDF‐15 levels are elevated systemically in both KPC mice and PDAC patients [20, 21], GDF‐15 binding to its receptor GFRAL on AP and NTS neurons induces anorexigenic and aversive responses, nausea, vomiting and delayed gastric emptying. Other signals may converge in these neuronal populations; for example, Interleukin (Il)‐6 is able to cross the blood brain barrier and activate neurons in the AP, NTS and PBN in the C26 model [22, 23], which manifests a highly Il‐6‐dependent cachexia. Il‐6 is not induced in the AP of mice with KPC cachexia [24].

Hypothalamic circuits that balance orexigenic and anorexigenic signalling seem at least partially intact in mice with KPC, despite profound reductions in overall intake. However, feeding responses were quantitatively blunted; feeding faltered after the first dark hour, and absolute intake following ghrelin or starvation was reduced. The hypothalamus is suggested to play a central role in cachexia, given its direct access to peripheral signals via its attenuated blood–brain barrier. KPC generates elevated systemic and central levels of a key mediator of satiation, lipocalin‐2 (LCN2), which may be related to these effects. LCN2 suppresses hunger by binding MC4R in the paraventricular nucleus of the hypothalamus; in KPC mice, LCN2 is predominantly produced by bone marrow and myeloid cells, readily crosses the blood–brain barrier, while genetic knockout of myeloid cell LCN2 in these mice substantially restores food intake, without altering tumour mass [25]. LCN2 is also elevated in patients with pancreatic cancer [21]. Beyond LCN2, inflammatory cytokines are likely participants in KPC cachexia. Hypothalamic inflammation is a key component of sickness behaviour, a coordinated series of changes in ingestion, locomotor activity, metabolism and other changes that reorganizes the organism's priorities to cope with infectious pathogens [26]. These effects are coordinated by proinflammatory cytokines through receptors on specific neuronal subsets (e.g.*,* tumour necrosis factor (TNF)α and interleukin (Il)‐1β directly inhibit the activity of AGRP neurons) [27]. Cancer cachexia resembles a prolonged sickness behaviour response [22, 26, 28]. KPC preferentially induces hypothalamic Il‐1β but not Il‐6 or TNFα [3, 24].

One goal of clinical cancer cachexia therapy is to restore food intake. The combination of blunted appetitive responses and excessive satiety has important therapeutic implications. We report blunted responses to ghrelin, as has also been reported in other cachexia models with disruption of ghrelin signalling by chronic inflammation and elevated cytokines [29]. In the MAC sarcoma model, daily intracerebroventricular AGRP injections produced transient increases in food intake, ending in unresponsiveness within 4 days, likely due to compensatory inhibitory input from upstream neurons or leptin signalling [30]. These findings highlight the limited efficacy of appetite‐inducing approaches in KPC, as these stimuli are effective only initially but are not sustained. Targeting of mechanisms that inhibit satiety may thus be a key part of an overall therapeutic approach. Inactivation of GDF‐15 by monoclonal antibody therapy largely blocks cancer‐associated weight loss in animal models, and a humanized monoclonal antibody has shown promising efficacy in Phase II clinical trials for anorexia in advanced stage cancer [14]. A small molecular weight antagonist of MC4R (TCMCB07) is effective in suppressing cancer‐ and chemotherapy‐induced weight loss in rodent models and is currently in Phase I/II clinical trials [12]. Ultimately, effective intervention may not require choosing between strategies to stimulate appetite or to counteract excessive satiety but may demand a combination of both approaches. Insofar as KPC seems to recapitulate some features of clinical pancreatic cancer cachexia, this model may prove useful in testing combinations of therapy simultaneously inhibiting MC4R and GDF‐15 (for example) to explore additivity or synergy of their actions.

### 4.1 Strengths and Limitations

Our studies were conducted under controlled conditions to minimize variability in murine feeding behaviour. Using CLAMS, we obtained high‐resolution, real‐time measures of meal structures and employed within animals to optimize the sensitivity of our behavioural measures. A key strength is our analytic framework: decomposing cancer‐associated ingestive behaviour into meal‐level parameters. Additional measures would enrich future studies of this type, for example, profiling appetite regulating factors and neurocircuits. Gastrointestinal dysmotility represents a plausible contributing mechanism to reduced food intake. Gastric emptying is suppressed in patients with cancer [31, 32]. Delayed gastric emptying is one of the effects of GDF‐15. Several physiological satiety peptides also have this action (cholecystokinin, glucagon‐like peptide‐1, peptide YY and amylin), and their effect is mediated via both humoral and vagal sensory neuronal pathways converging at the NTS. These specific processes were not directly measured here and represent an important focus for future work.

A limitation is that all measurements were performed in a single cancer model (KPC) at a defined disease stage. It remains to be investigated whether similar or distinct patterns of feeding parameters are perturbed in other cachexia models. Overall, these findings define the ingestive phenotype of KPC pancreatic cancer while providing a methodological and conceptual framework for broader comparative studies across models.

## Conflicts of Interest

The authors declare no conflicts of interest.

## Supporting information


**Table S1:** Summary of key anorexigenic and orexigenic mediators and neurons and their effects on meal parameters.
**Figure S1**: Change in daily food intake (relative to baseline) following the day of sham orthotopic (OT) ‐ and sham‐intraperitoneal (IP) procedure.
**Figure S2**: Evaluation of body weight and primary tumour mass 14 days post saline (sham) or tumour implantation (KPC) (n = 7/group).
