# Supplementary material for: Feeding Behaviour in the KPC Model of Pancreatic Cancer‐Associated Cachexia: Alteration of Spontaneous and Evoked Feeding Behaviours
Source: J Cachexia Sarcopenia Muscle. 2026 Apr 1;17(2):e70262. doi: 10.1002/jcsm.70262 (PMC13045440; doi:10.1002/jcsm.70262)
Supplement: Supplementary file 1 — Table S1: Summary of key anorexigenic and orexigenic mediators and neurons and their effects on meal parameters. Figure S1: Change in daily food intake (relative to baseline) following the day of sham orthotopic (OT) ‐ and sham‐intraperitoneal (IP) procedure. Figure S2: Evaluation of body weight and primary tumour mass 14 days post saline (sham) or tumour implantation (KPC) (n = 7/group). [file JCSM-17-e70262-s001.docx]

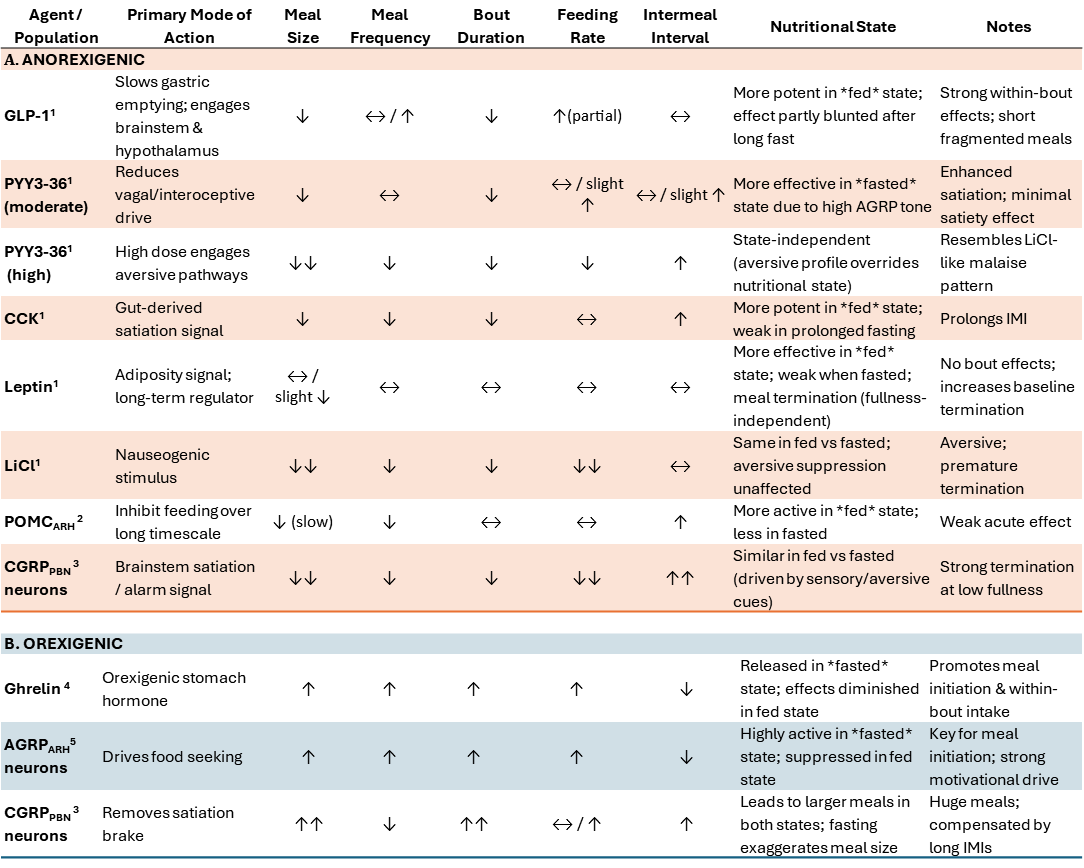
**Supplementary Table 1: Summary of key anorexigenic and orexigenic mediators and neurons and their effects on meal parameters.**

**The table summarizes key anorexigenic and orexigenic mediators and their directional effects on meal-level feeding parameters.** Physiological mediators (peptides/hormones) are shown based on effects demonstrated through exogenous administration, whereas neuronal populations are shown based on chemogenetic manipulations. Columns indicate the primary effects on; Meal Size, Meal Frequency, Bout Duration, Feeding Rate, and Intermeal Interval. Arrows represent changes in each parameter: ↑ increase; ↓ decrease; ↔ no change; combined arrows indicate context-dependent effects. Additional columns summarize modulation by nutritional state (fed vs. fasted) and provide brief mechanistic notes. Superscripts denote supporting sources (listed below) and correspond to the mediator/neuronal population in each row. **Abbreviations**: PYY, peptide YY; GLP-1, glucagon-like peptide-1; CCK, cholecystokinin; LiCl, lithium chloride; CGRPPBN, calcitonin gene-related peptide neurons; POMCARH, pro-opiomelanocortin neurons; AgRP, agouti-related peptide neurons; ARH, arcuate nucleus; PBN, parabrachial nucleus.

**Sources:**

¹ McGrath TM et al., PLOS Biology 2019.

² Zhan C et al., J Neurosci 2013.

³ Campos CA et al., Cell Metab 2016.

⁴ Teuffel P et al., J Physiol Pharmacol 2015.

⁵ Chen Y et al., eLife 2016.

**Supplementary Figure 1: Change in daily food intake (relative to baseline) following the day of sham orthotopic (OT) - and sham-intraperitoneal (IP) procedure.** Food intake was transiently reduced in sham-OT mice during the first 3 days post-procedure, consistent with effects of surgery and analgesics. Data are shown as mean ± SEM. *p < 0.05, **p < 0.01 by unpaired two-tailed t-test.

**B**

**A**

**Supplementary Figure 2: Evaluation of body weight and primary tumour mass 14 days post saline(sham) or tumour implantation (KPC)(n=7/group). (A) Body-weight measurements** were normalized to each animal’s Day 0 weight and plotted as percent change across the 14-day study. **Sham mice** started at 26.9 ± 1.4 g and increased to 27.6 ± 1.3 g, corresponding to an absolute gain of +0.65 ± 0.58 g (+2.4% relative to baseline). **KPC mice** started at 26.8 ± 0.8 g and showed an absolute body weight change of -1.98 ± 0.2 g over 14 days (-7.3% relative to baseline). Body-weight values at day 14 include tumour mass in the tumour-bearing group. **(B) Quantification of pancreatic mass** in sham vs KPC tumor-bearing mice at endpoint day 14. For the sham weight is of healthy pancreas and for KPC group, the mass represents the combined, weight of the pancreas and tumor. Data are presented as mean ± SD. *** p < 0.0001 by unpaired two-tailed t-test. These are the same animals described in main text Figure 3 & 5.
